# Fabrication of Artificial Leaf to Develop Fluid Pump Driven by Surface Tension and Evaporation

**DOI:** 10.1038/s41598-017-15275-y

**Published:** 2017-11-07

**Authors:** Minki Lee, Hosub Lim, Jinkee Lee

**Affiliations:** School of Mechanical Engineering, Sungkyunkwan University, Suwon, Gyeonggi-do 16419 Republic of Korea

## Abstract

Plants transport water from roots to leaves via xylem through transpiration, which is an evaporation process that occurs at the leaves. During transpiration, suction pressure is generated by the porous structure of mesophyll cells in the leaves. Here, we fabricate artificial leaf consisting of micro and nano hierarchy structures similar to the mesophyll cells and veins of a leaf using cryo-gel method. We show that the microchannels in agarose gel greatly decrease the flow resistance in dye diffusion and permeability experiments. Capillary tube and silicone oil are used for measuring the suction pressure of the artificial leaf. We maintain low humidity (20%) condition for measuring suction pressure that is limited by Laplace pressure, which is smaller than the water potential of air followed by the Kelvin-Laplace relation. Suction pressure of the artificial leaf is maximized by changing physical conditions, e.g., pore size, wettability of the structure. We change the agarose gel’s concentration to decrease the pore size down to 200 nm and add the titanium nano particles to increase the wettability by changing contact angle from 63.6° to 49.4°. As a result, the measured suction pressure of the artificial leaf can be as large as 7.9 kPa.

## Introduction

When small amount of water is to be transported from a reservoir to a target, mechanical and chemical pumps are generally necessary to generate pressure. Most common micropumps operate on fundamental principles such as piezoelectric^1^, thermopneumatic^2^, ultrasonic^3^, and electro-osmosis^4,5^. They require electric power or external energy to operate them and are composed of complicated and expensive components. Also, they are not very portable because of required electric input. Therefore, research on powerless pump has been continuously conducted for a long time to overcome these limitations. A surface tension driven pump is one of the types of powerless pumps^6–12^. This pump represents an attractive opportunity for its usage in microfluidics, since a high pressure difference can be generated by applying the Young–Laplace equation on the micro- and nanoscales (rather than the macroscale)^13^. Therefore, surface tension driven pumps are commonly used in lab-on-a-chip applications^8,10^. Evaporation driven pumps make use of surface tension on the surface and evaporation at the air-water interface^11,14–17^. Water transport is governed by the evaporation rate and flow resistance inside the devices.

The leaf is a well-known, natural, evaporation driven pump that operates through transpiration^18^. Many researchers have investigated the mechanisms for transport of water in trees and plants^19–22^. The suction pressure during transpiration in the leaf decreases below the atmospheric pressure; the maximum suction pressure can reach as low as −1.0 MPa^23^. Noblin *et al*. investigated optimal vein density using Polydimethylsiloxane (PDMS) channels that mimicked the xylem of the leaf and compared that with a real leaf during the evaporation^24^. Wheeler and Stroock fabricated a synthetic tree using an evaporative membrane to generate suction pressure and measured its flow rate^23^. Li *et al*. developed a micropump based on water potential difference using agarose gel sheets, which have nanopores^25–27^. In order to control the evaporation rate, they mimicked the stomata’s function; the flow rate of the artificial leaf could be controlled by shape of the artificial stomata. Zhou *et al*. fabricated continuous powerless pump using hydrophilic sponges which is operated by capillary action and evaporation. This pump was easy to use especially in microfluidic devices^28^. Kim *et al*. fabricated a stomata inspired membrane using a temperature responsive hydrogel, which can be used as an operative valve^29^. Most recently, the evaporative suction pressure quantifying device mimicking plant has been developed and pressure generating mechanism was reported^11,23^. Although, they have explained the pumping mechanism and its mimicking clearly, yet the fabrication method used for the development of their microfluidic device is somewhat complicated.

In this paper, inspired by the tree’s water transport system, we produce an artificial leaf using simple cryogel freezing method, which monolithically integrates the flow channel with an evaporation driven pump^30–32^. We develop an experimental setup which uses a glass capillary tube for measuring the precise suction pressure of the artificial leaf during evaporation. The suction pressure value indicates the pumping power transporting the water, which is generated by artificial leaf during the experiment. Also, the larger suction pressure in the artificial leaf was achieved by decreasing the flow resistance and varying parameters such as wettability and pore size.

## Results and Discussion

**Figure 1:**
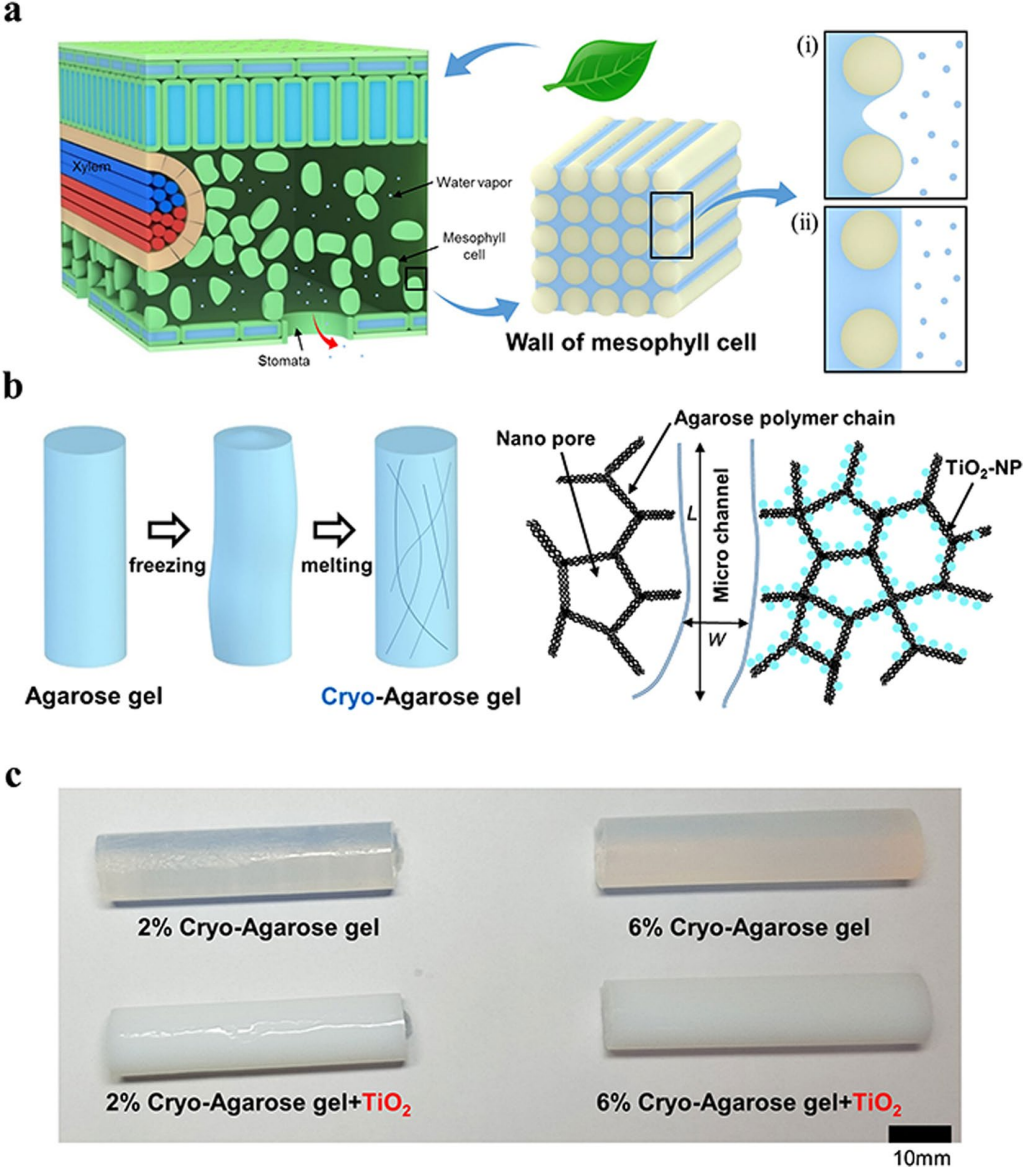
(**a**) Schematic of leaf structure and transpiration process in mesophyll cell wall. (**b**) Cryogel method which forms microchannel and schematic of nano-microstructure in agarose gel with TiO_2_ nanoparticle. (**c**) Four different cryo-agarose gels (2% and 6% agarose gels, with and without TiO_2_ nanoparticles).

Plants transport water from their roots to leaves via xylem through transpiration, which is an evaporation process that occurs in leaves. As shown in Fig. 1a, water transported via the xylem permeates to the mesophyll cell, which consists of microfibers^18^. At the wall of the mesophyll cell, an air-water interface exists between the fibers and evaporation occurs by controlling the transpiration flux through the opening and closing of stomata. As the water evaporates from the mesophyll cell, the evaporation rate of case (i) (Fig. 1a) is higher than that of case (ii). This comes from the large vapor pressure of thin water films existing on hydrophilic microfiber. This convex curvature of water film on fiber has large vapor pressure following the Kelvin-Laplace equation $\mathrm{ln}(\frac{p}{{p}_{0}})=\frac{2\sigma {v}_{m}cos\alpha }{rRT}$, where *p* is actual vapor pressure, *p*_0_ is saturated vapor pressure, *σ* is surface tension, *v*_*m*_ is the molar volume of the liquid, *R* is universal gas constant, *r* is radius of curvature which is positive for convex curvature and negative for concave curvature, *α* is contact angle and *T* is temperature^33^. Along with vapor pressure increase by convex curvature, case (i) has concave air-water interface between the fibers, generating pressure to pull the water continuously from the roots to the leaves via xylem, which is limited by Laplace pressure. On the other hand, the case (ii) is limited by diffusion which is acting on the air-water interface.

Inspired by the pumping mechanism of the leaf, we developed an artificial leaf with agarose gels, which consist of polymer chains similar to the mesophyll cell fibers. Four different agarose gels were prepared: 2 wt% and 6 wt%, with and without TiO_2_ nanoparticles. These parameters were chosen to investigate the effects of nanopore size and wettability. The mean diameters of the 2% and 6% agarose gel were 600 nm and 200 nm, respectively^34,35^. To make the water channel similar to the vein of a leaf, the cryogel method was used to produce randomly shaped microchannels, which decrease flow resistance in the agarose gel. The detailed method for producing the artificial leaf is described in the Method Section. We produced a cylindrical agarose gel with a microchannel length *L*_*m*_ and width *W*_*m*_. The TiO_2_ nanoparticles were attached on the polymer chains, as shown in Fig. 1b. As shown in Fig. 1c, the four cylindrical shaped agarose gels have the same diameter, 1.15 cm, and height, 5 cm.

**Figure 2:**
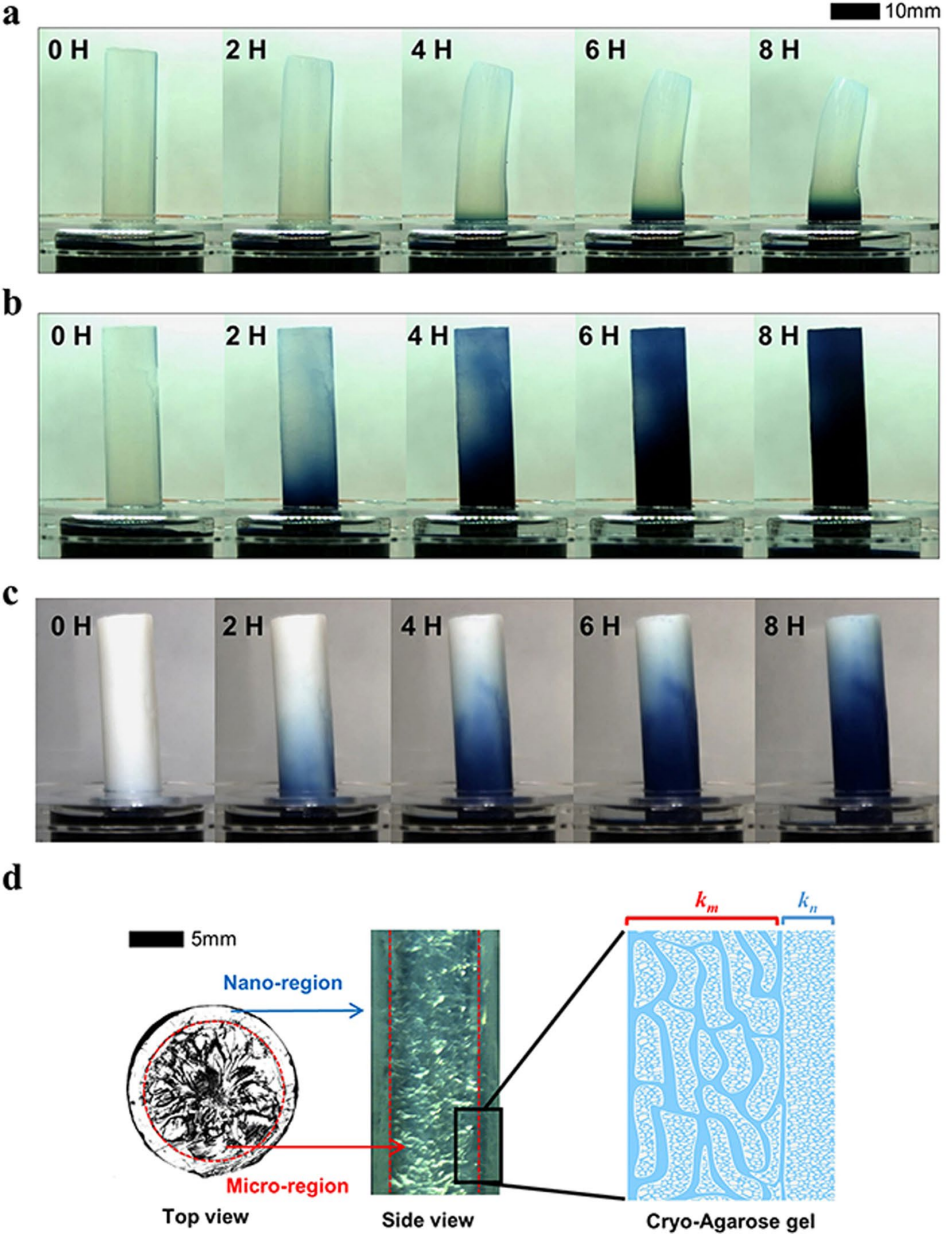
During the experiment (**a**) 2% agarose gel, (**b**) 2% cryo-agarose gel, and (**c**) TiO_2_ dispersed 2% cryo-agarose gel showed different diffusion and shrinkage. (**d**) Cross section and side view of cryo-agarose gel with nano- and micro-channels.

In order to test the pumping performance, the 2% agarose gel, 2% cryo-agarose gel, and TiO_2_ nanoparticle dispersed 2% cryo-agarose gel were exposed to air, except the bottom part which was dipped in water mixed with blue dye (PARKER, QUINK blue-black). During the experiments, the temperature and humidity were maintained at 20 °C and 20 ± 2%, respectively, and evaporation occurred from the agarose gel surface that was exposed to air. Because the water was barely transported from reservoir into the gel, the 2% agarose gel gradually shrank as times passed, and blue dye was transported only by diffusion through the nanopores, as shown in Fig. 2a. However, the 2% cryo-agarose gel and TiO_2_ nanoparticle dispersed 2% cryo-agarose gel (Fig. 2b and c) transported the blue dye via the microchannels by convection as well as diffusion. From the Fig. 2b and c, the dye height for 2% cryo-agarose gel and TiO_2_ nanoparticle dispersed 2% cryo-agarose gel apparently show some difference. To analyze this difference in both cases, the agarose cylinders were cut in half along the longitudinal axis (Figure S2 in Supporting Information). The internal cross-sectional views show that the blue dye has risen to the same height for both agarose cases. Thus, this apparent difference is because of the opaque nature of the TiO_2_ nano particle dispersed agarose gel which makes it almost impossible to see the actual height inside and therefore, the permeability for both cases should be similar which is also confirmed by the permeability measurements (Supporting Information Figures S4 and S5c). We believe that the TiO_2_ nano particles act to increase yield stress^36^ and reduce the speed of crystallization of ice and its growth^37^ without any effect on permeability (Supporting Information Figure S3). The cross section of the 2% cryo-agarose gel was captured by CCD camera (Manta, MG 282 C IRC), as shown in Fig. 2d. The cross-sectional images consist of nano- and micro-regions, which were easily distinguishable. Therefore, the cryo-agarose gel had a high transport rate because of the micro-region as shown in Fig. 2d.

To analyze the flow mechanism, we developed a theoretical model, which defines the permeability of the micro-region as *k*_*m*_ and the nano-region as *k*_*n*_. Using Darcy’s law, the flow resistance depending on permeability is described as follows:

1 $${\rm{R}}=\frac{\mu {\rm{L}}}{{\rm{2}}\pi {{\rm{r}}}_{{\rm{1}}}^{{\rm{2}}}{{\rm{k}}}_{{\rm{m}}}}+\frac{\mu {\mathrm{ln}(r}_{{\rm{2}}}{/r}_{{\rm{1}}})}{{\rm{2}}\pi {{\rm{k}}}_{{\rm{n}}}{\rm{L}}}$$

where *r*_1_ (4.73 mm) and *r*_2_ (5.75 mm) are the radii of the micro- and nano regions, *L* (5 cm) is the length of the agarose gel, and *μ* is viscosity of water (details about the derivation can be seen in the Supporting Information). According to Equation 1, the higher the micro-region permeability, the lower the flow resistance, as shown in Figure S5c. When we calculated the flow resistance by measuring the permeability of the agarose gel, the flow resistance of the 2% agarose gel was 2.7 × 10^15^ Pa·s/m^3^, and that of 2% cryo-agarose gel was 10^14^ Pa·s/m^3^. These results demonstrate that the development of the microchannels in agarose gel facilitates water flow by decreasing permeability to the surface from the reservoir. Therefore, cryo-agarose gels maintained their shape from evaporation during the experiments. While a leaf can control suction pressure through the stomata, which adjusts its evaporation rate, the evaporation rate of a cryo-agarose gel is fixed by constant temperature and humidity. Because of the low relative humidity (20%) in the controlled chamber, suction pressure of pumping device is limited by the meniscus at the nanopore surface which can be explained by Kelvin-Laplace relation. Because the Laplace pressure $({\psi }_{ext}=-\frac{2\sigma {\cos }\,\alpha }{r}=-0.9\,{\rm{MPa}})$ is around 230 times smaller than water potential $({\psi }_{ext}=\frac{RT}{{v}_{m}}\,\mathrm{ln}(RH)=\,-217\,\mathrm{MPa})$, the meniscus quickly changes its curvature from flat to curved surface alike the schematics of (i) and (ii) of Fig. 1a.

**Figure 3:**
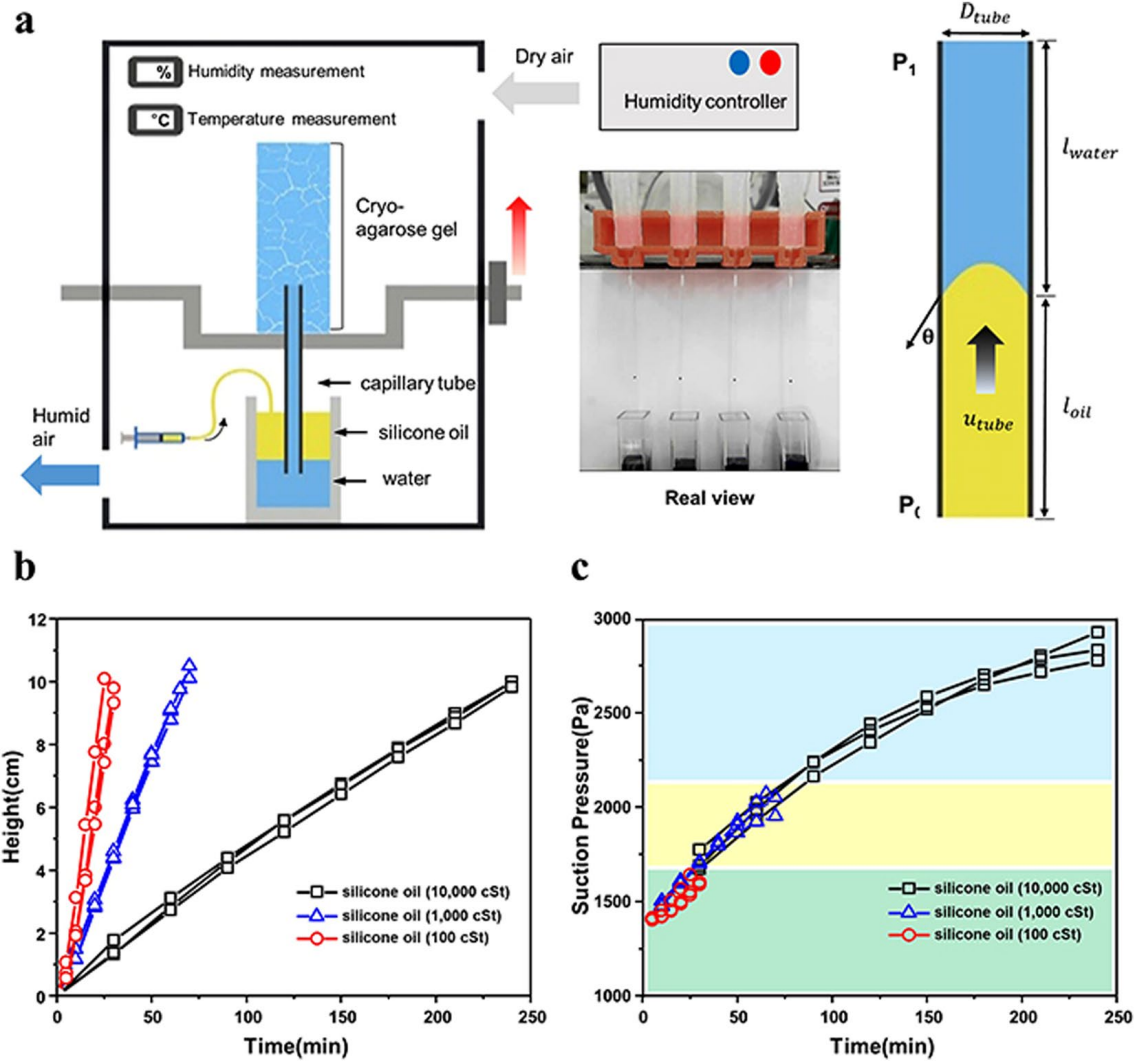
(**a**) Schematic of experimental setup for measuring the suction pressure. During the experiment, the position of the oil/water interface was measured to calculate performance. (**b**) Measured position of the oil/water interface in capillary tube, and (**c**) calculated pressure using several silicone oils.

At the bottom of the cryo-agarose gel, a glass capillary tube (inner diameter of 346 μm and length of 12 cm) was connected to observe the fluid flow and measure the velocity as the fluid transported from reservoir to cryo-agarose gel. The reservoir had two layers: water mixed with blue dye and silicone oil. We used three different silicone oils (100, 1000 and 10000 cSt, Shin-Etsu Silicone, KF-96H) to vary the flow resistance in the capillary tubes. For the first step in the experimental procedure, the end tip of the capillary tube was immersed into the water. The water flowed through the capillary tube because of the suction pressure generated by the artificial leaf. Once the artificial leaf pulled the water from the reservoir, we raised the capillary tube using jig so that the end position of it meets the oil layer. Once the oil flowed, the oil/water interface was observed in the capillary tube. The meniscus position was captured by CCD camera during the experiment (Fig. 3a and Supporting Movie 1). The position of the interface was measured as a function of time (Fig. 3b and Supporting Movie 1). Depending on the silicone oil viscosity, the velocities of the interface were different. The generated pressure by artificial leaf was calculated by the following equation:

2 $$\Delta {P}={U}\times (\frac{{32}{{\mu }}_{{water}}{{L}}_{{water}}}{{{D}}^{{2}}}+\frac{{32}{\mu }_{{oil}}{{L}}_{{oil}}}{{{D}}^{{2}}})+{g}\times ({\rho }_{{water}}{{L}}_{{water}}+{\rho }_{{oil}}{{L}}_{{oil}})+\frac{{4}\sigma \,{\cos }\,{\alpha }}{{D}}$$

This equation describes the effects of viscosity, gravity, and surface tension acting inside the capillary tube on the driving pressure, where *L*_*water*_ and *L*_*oil*_ are the heights of the liquid in capillary tube, *D* is tube diameter and *ρ* is density. Surface tension and the contact angle at the oil/water interface were measured as 36 mN/m and 37°, respectively (Supporting Information Figure S9). The suction pressures in the capillary tube was calculated by Equation 2 and plotted as shown in Fig. 3c. They were in different ranges, depending on the viscosity of the silicone oils (100, 1000 and 10000 cSt). The high suction pressure can be measured when we used high viscosity silicone oil (10000 cSt). During the suction, we believe that the air/water meniscus on the agarose surface changed its contact angle and moves into agarose gel since the water potential is a lot larger than Laplace pressure.

**Figure 4:**
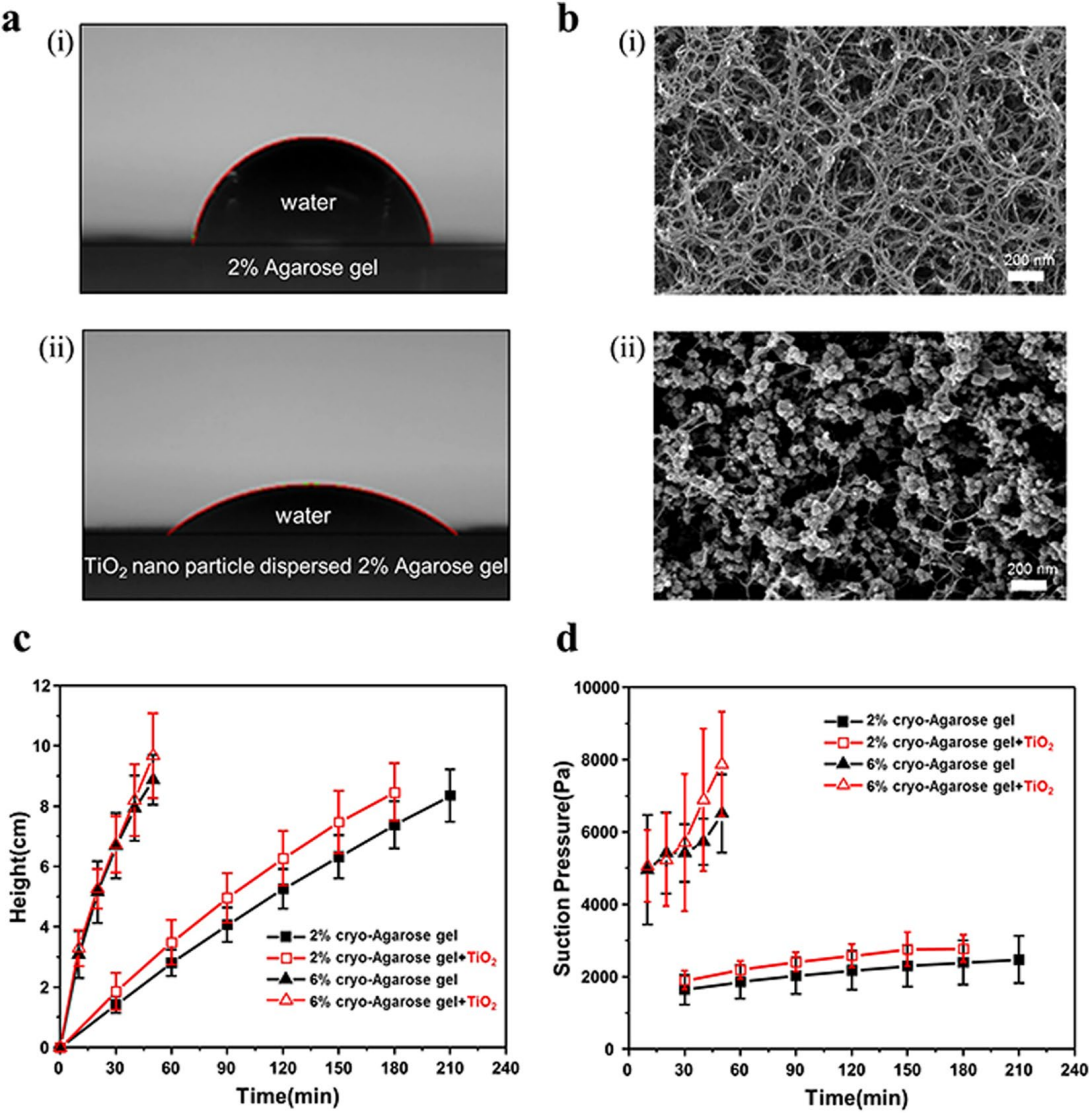
(**a**) Dried TiO_2_ nanoparticle dispersed agarose gel has lower contact angle than dried agarose gel. (**b**) SEM images, 2% agarose gels and TiO_2_ nanoparticle dispersed 2% agarose gel. (**c**) The meniscus position during fluid pumping. (**d**) The calculated suction pressure of 2% and 6% agarose gels, with and without TiO_2_ nanoparticles.

Moreover, we investigated the method to increase the suction pressure of the agarose gel. In our experiments, the radius of the nanopores and the contact angle was varied. The contact angle could be changed by increasing wettability (i.e., adding TiO_2_ nanoparticles to the agarose solution before gelation). The contact angles were measured using dried agarose gel and Smartdrop (Femtofab, SDL200TEZD), as shown in Fig. 4a. When the agarose gel was dried out at room temperature (Supporting Information S7), its nano fibers structure was collapsed by the surface tension of water between the fibers during the drying process and had a very small permeability. One the other hand, agarose nano fiber structure was retained during critical point drying (CPD, Leica-EM CPD300). In Fig. 4a, the contact angles of the dried agarose gel and the TiO_2_ nanoparticle dispersed agarose gel were 63.6 ° (±3.6°) and 49.4 ° (±2.9°), respectively. In order to observe the agarose gel fiber structure, images of the CPD dried 2% agarose gel and the TiO_2_ nanoparticle dispersed 2% agarose gel were captured by FESEM (JEOL, JSM-7500F). TiO_2_ nanoparticles were attached to the agarose nanofiber as shown in Fig. 4b and schematically shown in Fig. 1b.

The suction pressure of TiO_2_ nano particle dispersed 2% cryo-agarose gel (only wettability change) is 1.08 times larger than 2% cryo-agarose gel. And, the suction pressure of TiO_2_ nano particle dispersed 6% cryo-agarose gel (wettability + pore size change) is 2.8 times larger than 2% cryo-agarose gel. Thus, maximum performance can be achieved by changing the wettability and pore size together. The graph in Fig. 4c shows changing meniscus position with respect to time. The error bars in the graph indicate the fact that the agarose gels produce random shaped microchannels because of the variable ice crystal growth that consequently, influences the suction pressure. The TiO_2_ nanoparticle dispersed 6% cryo-agarose gel had the highest suction pressure (7.9 kPa), as shown in Fig. 4d, which was calculated by Equation 2. Although it confirms a large value of suction pressure, yet it did not show the steady state due to length limit of glass capillary, unfortunately.

## Conclusions

A leaf inspired pump was developed based on transpiration. A plant leaf is a high-efficiency hydraulic pump composed of porous materials. By mimicking a plant leaf, a leaf inspired pump was produced with cryo-agarose gel, which consists of hierarchical regions of random microchannels and nano sized agarose gel’s polymer chains. The microchannels mimic the vein of the leaf, decreasing flow resistance in agarose gel, and the agarose gel’s polymer chain mimics the mesophyll cells, generating the suction pressure. In this study, since we conducted the experiments at constant low humidity where the suction pressure is limited by Laplace pressure, we focused on the pumping pressure and its dependence on wettability and pore size which is possible to be explained by Kelvin-Laplace equation $\mathrm{ln}(\frac{p}{{p}_{0}})=\frac{2\sigma {v}_{m}cos\alpha }{rRT}$. The results indicated that these two parameters critically affect the pumping pressure, and is explained by theoretical description. When we increase the wettability of the agarose gel by using TiO_2_ nanoparticles, it affects the contact angle of the agarose fiber, increasing the suction pressure. Decreasing the agarose gel pore size also increased suction pressure. Through the experiments, we calculated the precise suction pressure of the leaf inspired pump. Even though our leaf inspired pump could not pump like a tree, this study could lead to the development of a novel hydraulic pump in the future.

## Methods

### Preparation of the artificial leaf

To make normal agarose gel, 2 g or 6 g of agarose powder (Agarose-LE, Affymetrix Inc., USA) was put into a beaker with 98 g or 94 g of deionized water. To make TiO_2_ added agarose gel, 0.2 g of TiO_2_ nanoparticles (Diameter was 21 nm, Degussa P25) were added to the solution and the solution was sonicated for 30 min. Next, these solutions were heated until the agarose powder was dissolved. Once it dissolved, the solution was poured into a cylindrical shaped mold. After gelation, the prepared agarose gels were stored in deionized water to prevent drying. To make a cryo-agarose gel, the agarose gel was frozen at −35 °C for 1 hr to allow the water molecules to form coarse ice clusters. (Supporting Information) Then, the frozen agarose gel was stored in DI-water, the ice clusters melted, and the microchannels were randomly shaped in the agarose gel.

### Critical Point Drying (CPD) method for SEM image capture

In order to capture the SEM image, the agarose gel was treated by the CPD method, which removes the liquid in porous media without any deformation. The agarose gel can deform during evaporation because surface tension works on the agarose structure. Before using the CPD method, the water in the agarose gel was replaced by ethanol through a dehydration process. Then, using the CPD machine (Leica, EM CPD300), all the liquid in the agarose gel was removed, and only the agarose gel polymer structure remained. Finally, after coating the sample with platinum, the SEM images can be captured by FESEM (JEOL, JSM-7500F).

### Preparation of dried agarose film

To compare the contact angles of the agarose gel and the TiO_2_ nanoparticle dispersed agarose gel, 1 mm thick sliced gels were prepared and dried at room temperature. Consequently, contact angles were measured by Smartdrop (Femtofab, SDL200TEZD).

## Electronic supplementary material

supplementary information

Artificial Leaf Oil Suction Movie File
