# Supplementary material for: Fabrication of Artificial Leaf to Develop Fluid Pump Driven by Surface Tension and Evaporation
Source: Sci Rep. 2017 Nov 7;7:14735. doi: 10.1038/s41598-017-15275-y (PMC5676738; doi:10.1038/s41598-017-15275-y)
Supplement: Supplementary file 1 — supplementary information [file 41598_2017_15275_MOESM1_ESM.pdf]

Supporting Information

Fabrication of Artificial Leaf to Develop Fluid Pump Driven by Surface Tension and Evaporation

Minki Lee[1], Hosub Lim[1] and Jinkee Lee[1,*]

[1]School of Mechanical Engineering, Sungkyunkwan University, Suwon, Gyeonggi-do 16419, Republic of Korea

*lee.jinkee@skku.edu

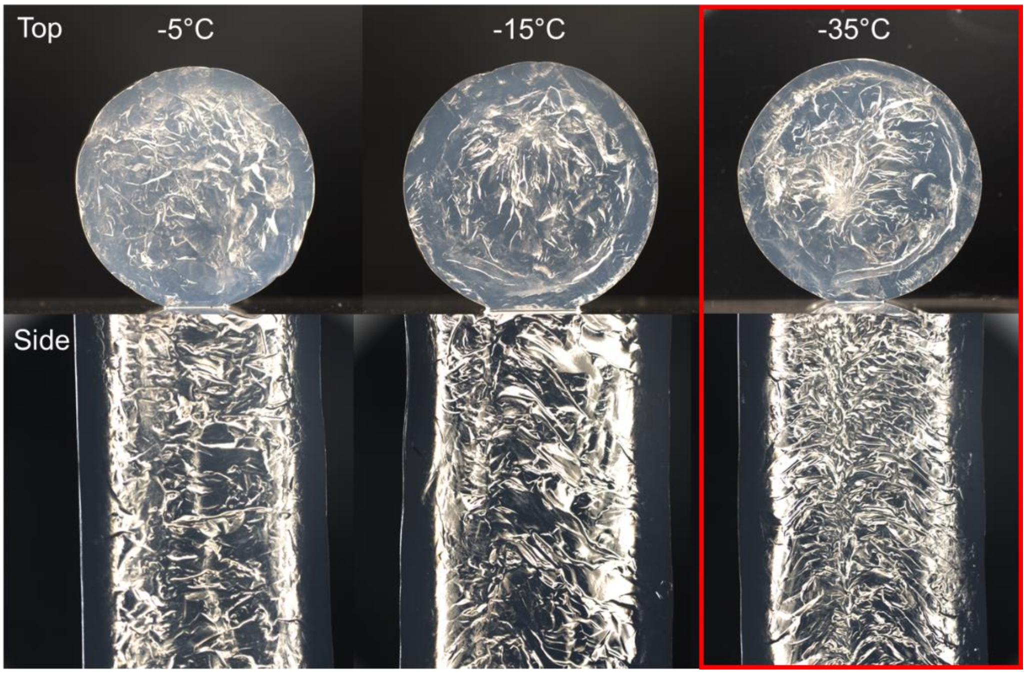

Figure S1. Microchannels formation depend on freezing temperature.

To make microchannels within agarose gel which is used in the experiments, cryo-gel freezing method at a fixed temperature of -35°C was used. To verify the temperature effect on microchannels fabrication, two additional frozen gels at -5 and -15°C were also investigated. As shown in Figure S1, the channel density inside the agarose gel varies depending on freezing temperatures. The lower the temperature, the denser are the microchannels and the shorter is the time required for their fabrication.

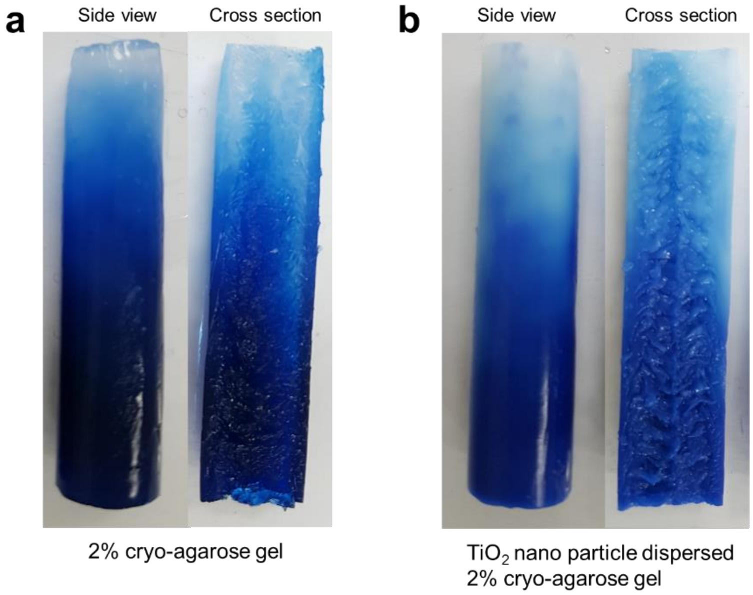

Figure S2. Side view and cross section view of dye diffused (a) 2% cryo-agarose gel and (b) TiO_2 nanoparticle dispersed 2% cryo-agarose gel.

The dye experiment shows the increase of the water transport efficiency through micro-channels in different agarose gels. In Figure 2 of the manuscript, the water transport for agarose gels with and without TiO_2 nanoparticles apparently show different heights. To confirm whether or not the water transport height is different, we cut the agarose cylinder in half along the longitudinal axis and the image taken is as shown in Figure S2. The results show that the dye height for both agarose gel cases is similar and it is the opaque nature of agarose gel with TiO_2 nanoparticles which makes it almost impossible to see actual height inside. The same height level indicates that the permeability in both cases should be similar which is also confirmed by the permeability measurement as shown in Figure S5.

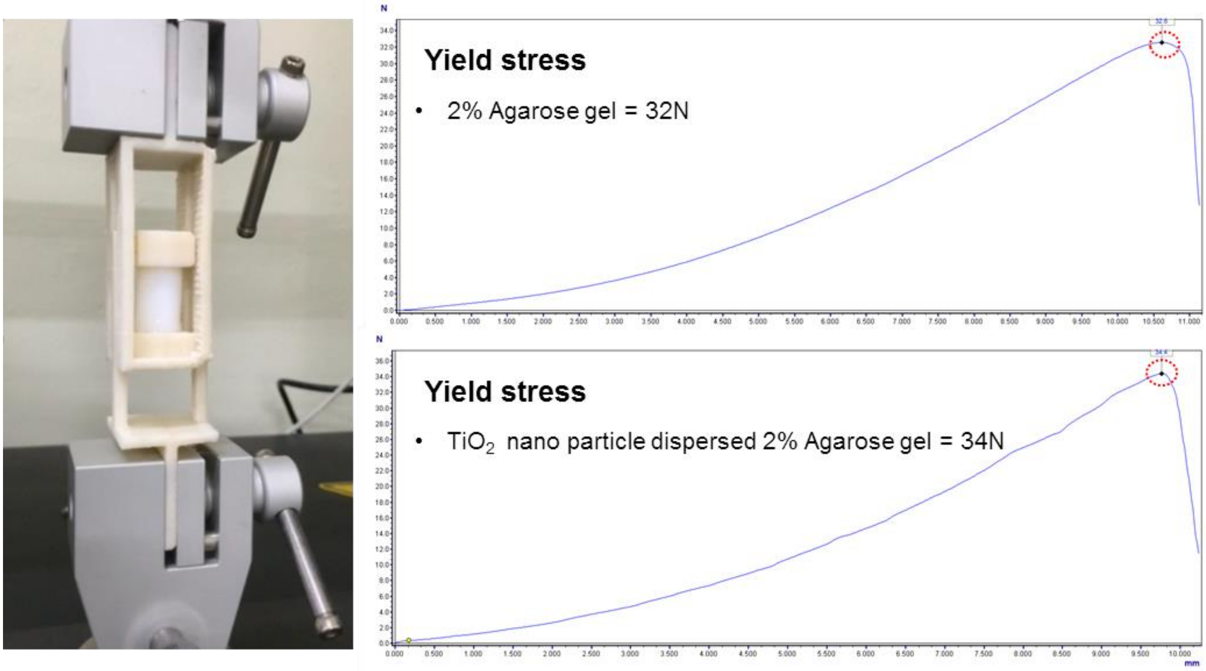

Figure S3. Measuring the yield stress of 2% agarose gel and TiO_2 nanoparticle dispersed 2% agarose gel.

To measure the yield stress of agarose gels, we conducted tensile tests on a Tensile Testing Machine (QC-506M2, Cometech Testing Machines, Taichung, Taiwan) using a 200 N load cell; the yield stresses of the agarose gels were measured as shown in Figure S3. TiO_2 nanoparticle dispersed 2% agarose gel had a higher yield stress than 2% agarose gel, because the TiO_2 nanoparticle increase the yield stress of the polymer (agarose).

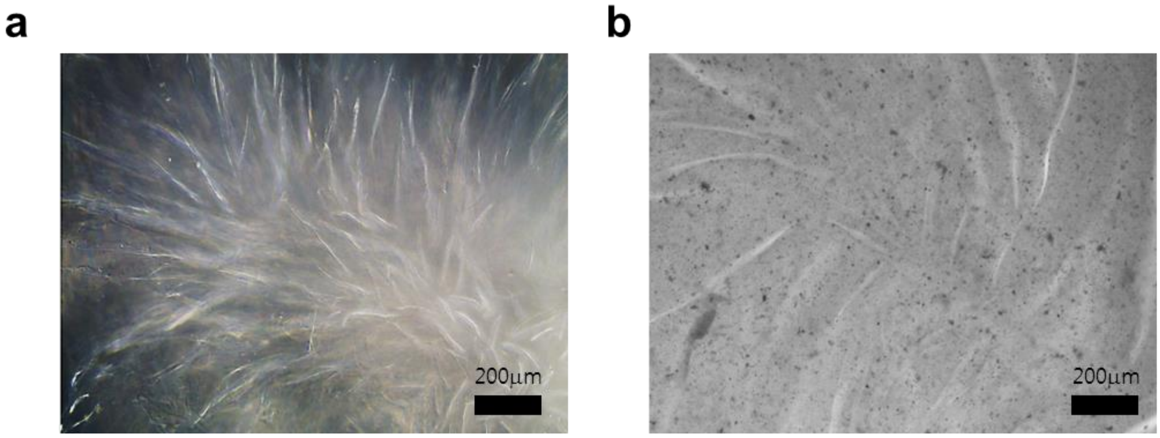

Figure S4. Top view of (a) 2% cryo-agarose gel and (b) TiO_2 nanoparticle dispersed 2% cryo-agarose gel.

When we observed the agarose gels using inverted microscope (Nikon, ECLIPSE Ti-U), the different shapes of microchannels were formed because of TiO_2 nanoparticle.

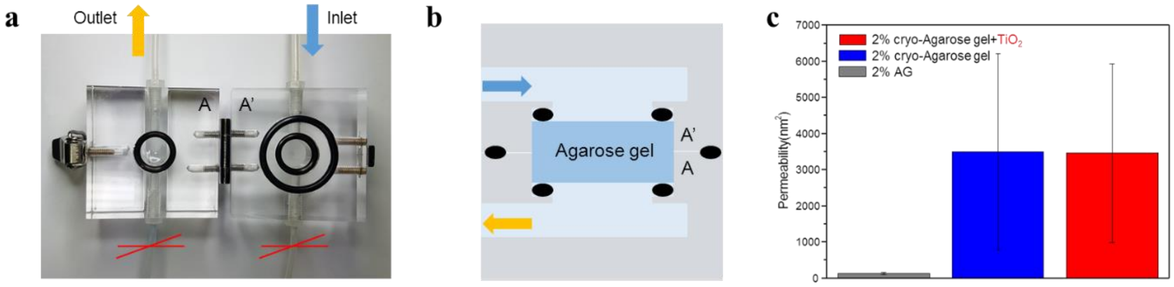

Figure S5. Measuring permeability (a) Permeability measurement system. (b) Schematic side view of measuring system. (c) Permeability of each sample.

We developed the permeability measurement system using risen transparent acryl and an O-ring, which is used to prevent water leaking. Firstly, we prepared a new sample (with diameter of 20 mm and height of 8 mm) for measuring the permeability. Secondly, the sample was placed between small O-rings, as shown in Figure S5b. We produced constant pressure (1 kPa) at the inlet with a microfluidic flow control system (FLUIGENT, MFCS-EZ). At the same time, we measured the mass flux using a precision balance (OHAUS, Explorer EX224G). Finally, the permeability of sample was calculated by Darcy's law. The permeabilities were as follows: 2% agarose gel was 132 nm^2, 2% cryo-agarose gel was 3502 nm^2, and TiO_2 nanoparticle dispersed 2% agarose gel was 3462 nm^2. As shown in Figure S5c, the permeability of the cryo-agarose gel had a large variation since the cryo-method randomly produces microchannels in agarose gel during freezing.

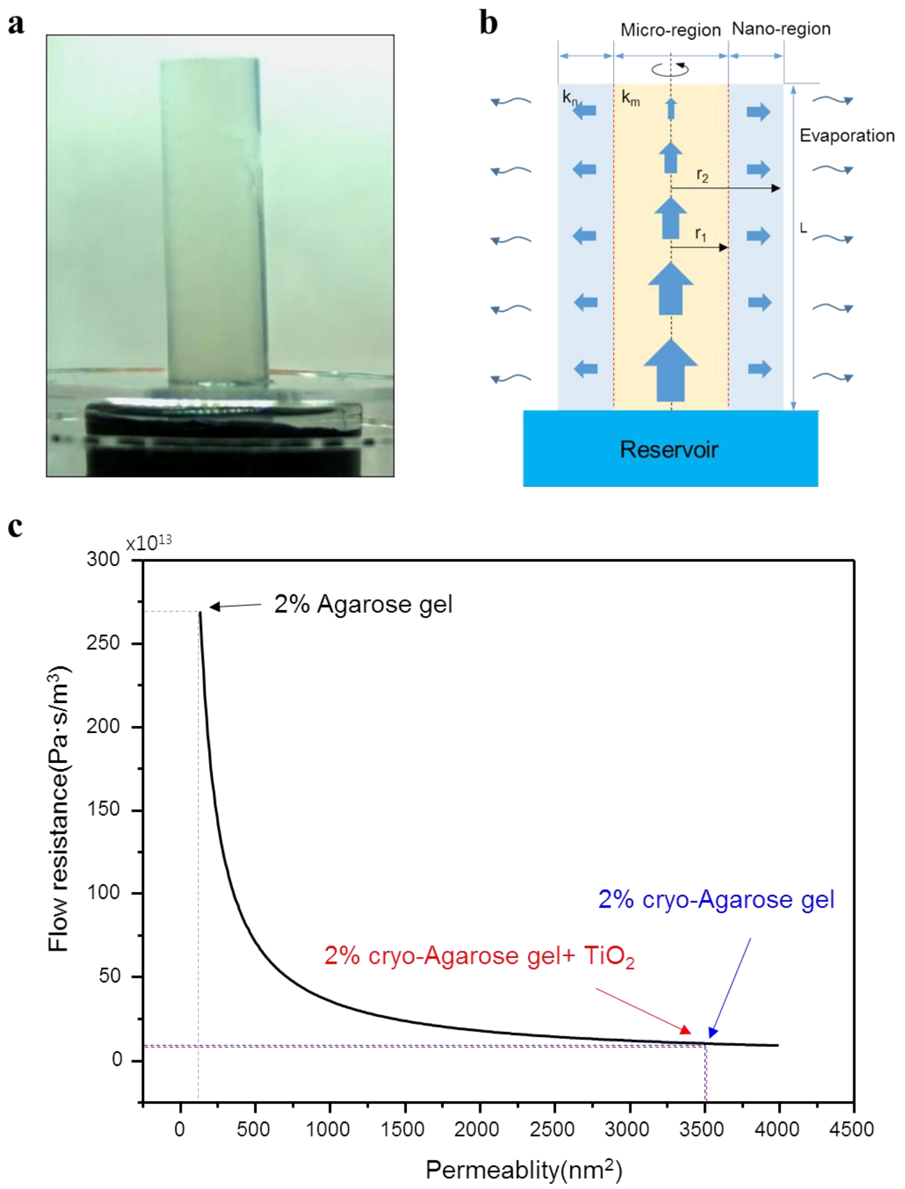

Figure S6. Flow resistance in artificial leaf (a) Pumping experiment for cryo-agarose gel. (b) Schematic of flow direction in cryo-agarose gel during the evaporation. (c) Flow resistance depending on permeability of micro-region.

Let $z = 0$ denote the base of the agarose gel and $z = L$ denote the top of the agarose gel. Then using mass balance, we can express the following:

$$A[u(z)-u(z+\Delta z)]-q\Delta z=0,$$

where u is the average water velocity of the fluid in the micro-region, $q\Delta z$ is the evaporation rate of the agarose gel, and A is cross-sectional area of the micro-region of the agarose gel. After the equation is divided by Δz and taking the limit of $\Delta z \to 0$, we arrive at the following:

$$\frac{du}{dz}=-\frac{q}{A}. \qquad (1)$$

Through Darcy's law and using the permeability of the micro-region (k_m), pressure and velocity have the following relationship:

$$\frac{dp}{dz}=-\frac{\mu u}{k_m}, \qquad (2)$$

where μ is the viscosity of water. Then, combining Equation 2 and 3, the equation for pressure becomes the following:

$$\frac{d^2p}{dz^2}=\frac{\mu q}{Ak_m}. \qquad (3)$$

The boundary condition becomes:

$$\begin{aligned}&\text{At } z=0: \quad p(0)=p_0\\ &\text{At } z=L: \quad p(L)=p_{atm} \quad u=0\end{aligned} \qquad (4)$$

We obtain the pressure equation, depending on the height of the agarose gel as follows:

$$p(z)=\frac{\mu q}{2Ak_m}(z-L)^2+p_{atm}. \qquad (5)$$

In the nano-region, we assume that fluid flows only in the radial direction, because the nano-region has low permeability, causing high flow resistance. Thus, Darcy's law in the radial direction is used as follows:

$$\frac{dp}{dr} = -\frac{\mu Q}{A_r k_n}, \tag{6}$$

where A_r is $2\pi rL$, and Q is total evaporation rate from the agarose gel. The above equation is integrated from r_1 to r_2. The result is as follows:

$$\Delta p = \frac{\mu Q ln(r_2/r_1)}{2\pi L k_n}. \tag{7}$$

When we add Equation 4 and 6, we obtain the total pressure difference that is necessary to pump the water from the reservoir to the surface of the agarose gel,

$$\Delta p = Q \times \left(\frac{\mu L}{2\pi r_1^2 k_m} + \frac{\mu ln(r_2/r_1)}{2\pi L k_n} \right). \tag{8}$$

We can represent the flow resistance of the cryo-agarose gel as follows:

$$R = \frac{\mu L}{2\pi r_1^2 k_m} + \frac{\mu ln(r_2/r_1)}{2\pi L k_n} \tag{9}$$

As shown in Figure S6c, the flow resistance rapidly decreases as the permeability of the microregion increases.

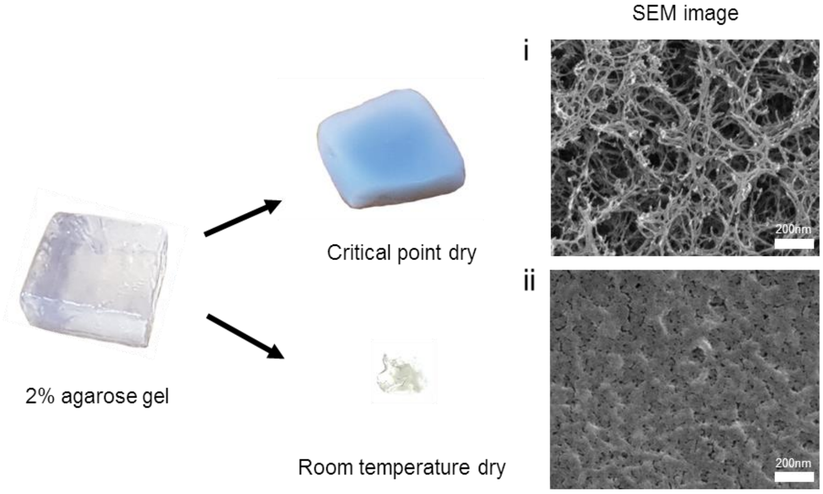

Figure S7. Comparing shape and nano fiber of SEM image between critical point drying and room temperature drying agarose gel.

To characterize the nano fiber inside agarose gel, the CPD (Critical Point Drying) method was used. The detailed experimental procedures are described in manuscript. When agarose gel is dried in room temperature, the surface tension pulls the nano fibers and reduced the volume. However, the CPD method makes it possible to dry agarose without volume reduction. The SEM images in Figure S7 shows the pore size change.

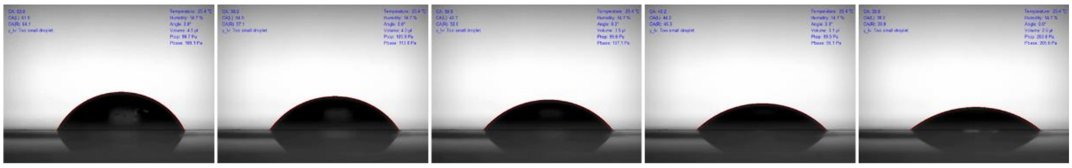

Time (min)	0	4	8	12	16
Contact angle (°)	63	56	50.8	45.2	39

Figure S8. Contact angle change on room temperature dried agarose gel versus time.

The sequential time images shows the contact angle measurement images for room temperature dried agarose gel, not CPD dried. Figure S8 shows the reduction of contact angle with time since the water is absorbed into porous agarose gel, although, the pore size reduced dramatically during drying. However, the speed of absorption is very slow and the contact angle change is as low as -1.5 °/min. The contact angle value in manuscript was measured within 10 seconds to minimize the measuring error and this contact angle change is negligible.

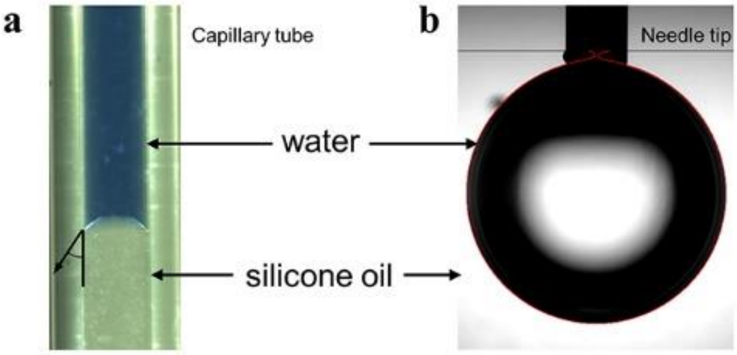

Figure S9. Measuring contact angle and interfacial tension (a) Contact angle between water and silicone oil in capillary tube. (b) Image of pendant-drop method for measuring the interfacial tension between water and silicone oil.

The inner diameter of the capillary tube is 346 μm. During the procedure for measuring the pumping pressure (Figure 3), the meniscus between the water and silicone oil is formed in the capillary tube. During the experiment, it gradually moved up while maintaining the contact angle at 37°, as shown in Figure S9a. In order to calculate the pumping pressure (Equation 2 in the main text), the interfacial tension was measured. The interfacial tension between the water and the silicone oil was 36 mN/m, which was measured by Smartdrop (Femtofab, SDL200TEZD) using the pendant drop method.

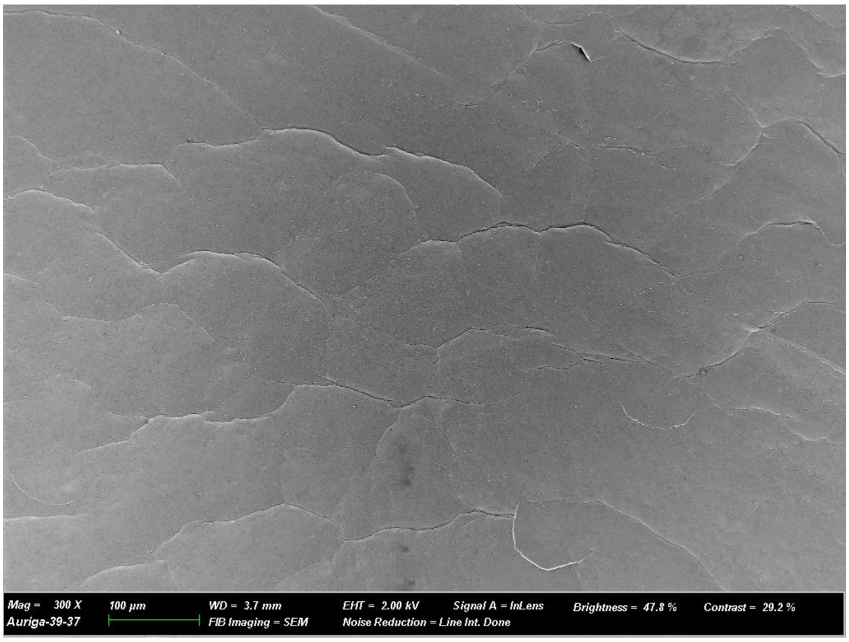

Figure S10. SEM image of microchannels on the 2% cryo-agarose gel surface.

Not only an optical microscope, but also SEM can observe the microchannels that are generated by the cryogel method. As we mentioned in manuscript, the microchannels are randomly formed on the surface.
